# Voluntary first responders’ experiences of being dispatched to suspected out-of-hospital cardiac arrest in rural areas: an interview study

**DOI:** 10.1186/s12872-024-03826-x

**Published:** 2024-03-14

**Authors:** Camilla Allert, Bengt Nilsson, Anders Svensson, Ewa K. Andersson

**Affiliations:** 1 Department of Health and Caring Sciences, University Lecturer Faculty of Health and Life Sciences Linnaeus University, 392 31 Växjö/Kalmar, Sweden; 2 Department of Forestry and Wood Technology, Linnaeus University, Växjö, Sweden; 3 Centre of Interprofessional Collaboration Within Emergency Care (CICE), Linnaeus University, Växjö, Sweden; 4 Department of Ambulance Service, Region Kronoberg, Växjö, Sweden; 5 Agunnaryd Voluntary Fire Brigade, Ljungby, Sweden

**Keywords:** Cardiopulmonary resuscitation, Experiences, Out-of-hospital cardiac arrest, Qualitative research, Voluntary first responder

## Abstract

**Background:**

Out-of-hospital cardiac arrest (OHCA) is a leading cause of death, and survival outcomes vary across countries and regions. To improve survival, the European Resuscitation Council Guidelines encourage the implementation of technologies like smartphone applications to alert voluntary first responders (VFRs) who are near a suspected OHCA. VFRs are of great importance in the ´chain of survival´, but there is still a lack of knowledge about their experiences; especially of those operating in rural areas. Understanding those experiences is crucial in developing appropriate interventions to train, encourage, and safeguard VFRs in their mission. Therefore, the aim of this study was to describe VFRs´ experiences of being dispatched to suspected OHCA in rural areas.

**Methods:**

The study used an inductive design. The data were collected using individual interviews with 16 VFRs and analysed using qualitative content analysis.

**Results:**

The results are presented in terms of six generic categories ‘‘Being motivated and prepared’’*,* ‘‘Having strategies to undertake the mission’’*,* ‘‘Collaborating with others’’*,* ‘‘Being ethically aware’’*,* ‘‘Supporting the family members’’*,* and ‘‘Coping with the mission’’*,* which formed the basis of the main category ‘*Desire to save lives and help others*’. The findings showed that VFRs had a genuine desire to contribute to save lives in this rural area. Regardless of the circumstances, they were prepared to leave everything and act to the best for the victim and their family members. In theirs’ missions they collaborated with others at the scene and were guided by ethics while they acted in complex circumstances.

**Conclusions:**

VFRs dispatched in rural areas express a desire to save lives. In their missions, they acted in complex situations and experienced both emotional and ethical challenges.

The design, implementation, and evaluation of support interventions directed at VFRs should be prioritised, especially in rural areas, as it can contribute to more people becoming and remaining VFRs, which in turn could contribute to sustainable development.

## Background

Out-of-hospital cardiac arrest (OHCA) is a leading cause of mortality, and survival outcomes vary substantially across countries and regions [1]. The chance of surviving OHCA depends on the efficiency of the ´chain of survival`, where each link is equally important [2, 3]. This chain now includes bystander cardiopulmonary resuscitation (CPR) as an essential component [4] while waiting for the arrival of emergency medical services (EMS). The European Resuscitation Council guidelines emphasise the importance of using technology such as text messages or smartphone applications to alert voluntary first responders (VFRs) to suspected OHCAs and improve the rate of bystander CPR [5]. This is important, as prolonged response time for EMS arrival is common [6, 7], and time to CPR significantly affects survival outcomes [8, 9]. In Sweden, the ambulance response time in the case of OHCA has significantly increased over time, from 5 min in the early 1990s to 12 min in 2020 [10]. Despite the significant improvement by 2022, ambulance response time is still 10 min on average [11] and just over 17 min in rural areas [7]. This is important, as short EMS response time is also vital in maintaining the benefits of bystander CPR [6].

Although it has been shown that bystander CPR plays a key role in improving OHCA survival [8, 12–14], there is still a need to increase bystander CPR rates [3]. In a comparison of rural and urban areas, it was shown in a nationwide study from Denmark that while it was more likely a patient would receive bystander CPR in rural areas, 30-day survival rates were still lower than in urban areas [15]. According to Malta Hansen [16], there are factors that facilitate a VFR to attempt bystander CPR and the use of an automated external defibrillator (AED). Those factors include, for example, previous knowledge that CPR is vital in enhancing survival, previous hands-on training in CPR and the use of AED, and feeling a moral obligation to act to the best of others and to work together as a team. Matsuyama et al. [17] identified fear of causing harm or making mistakes and lack of CPR knowledge and skills as barriers to the initiation of bystander CPR and willingness to respond to OHCA. A lack of prior CPR exposure and emotional barriers such as anxiety and hysteria were also identified as barriers to act. Providing bystander CPR could be experienced as emotionally challenging and may cause uncertainty and concern that could prove difficult to face in daily life [18]. A recent systematic review [19] confirmed that psychological and behavioural factors had impact on the CPR initiation. It has also been shown that even if most VFRs consider their CPR to have been a positive experience and would resuscitate again, they experienced severe short-term psychological impacts [20]. Participants in a study by Södersved Källestedt et al. [21] described that they were not always prepared for their own feelings in the unexpected situations they met with when dispatched to OHCA. It is recognised that the VFRs are of great importance in the ´chain of survival´, but there is still a lack of knowledge about their experiences [22], especially from VFRs who are dispatched to rural areas. Understanding these experiences is crucial to develop appropriate interventions to train, encourage, and safeguard VFRs in their mission. Therefore, the aim was to describe VFRs’ experiences of being dispatched to suspected OHCA in rural areas.

## Methods

### Design

An inductive research design was chosen to reach a deeper understanding of the participants’ inner perspectives [23]. The study is reported according to the Consolidated Criteria for Reporting Qualitative Research checklist [24].

### Context

The study was conducted in one region in a rural area in southern Sweden (area: 8500 km^2^, population: approaching 200,000 inhabitants, density 24 inhabitants/km^2^). This region was divided into eight municipalities varying between 8500–96000 inhabitants, and a population density between 8–54.7 inhabitants/km^2^. The region included two cities, even if some of the areas have several inhabitants and higher population densities, the actual region will be defined as a predominantly rural area according to urban–rural topology, including degree of urbanisation, which is the recommended method to use to define rural areas to facilitate international comparisons [25].

There were 17 Advanced Life Support (ALS) ambulances available in the region during the day on weekdays, of which 11 were available around the clock. Each ambulance was equipped with an AED, a Lund University Cardiopulmonary Assist System (LUCAS), and had at least one registered nurse (RN) on duty. When OHCA was suspected, two ambulances were dispatched. Each municipality in this region had a fire department (FD) staffed with full-time, part-time, or volunteer firefighters. All firefighters were trained in Basic Life Support (BLS), equipped with an AED, and dispatched as First Responders (FRs) to OHCA if there were not two ambulances available or if the FD was more likely to arrive first at the scene. In five of the eight municipalities, part-time crew managers acted as a First Incident Person (FIP) responding directly and arriving to the OHCA alone before the entire crew.

In this region, a smartphone GPS positioning system called SMS-lifesaver (developed by Heartrunner Sweden AB) for locating and alerting nearby VFRs to OHCA has been used since 2020 in addition to ambulance and FD dispatch. This SMS-lifesaver is currently available in 12 of the 21 regions in Sweden and is active from 7 a.m. to 11 p.m. By downloading the SMS-lifesaver application and registering, adult persons trained in CPR can volunteer as FRs to be located and dispatched (by emergency medical dispatch centre) to suspected OHCA. The dispatches do not include children below the age of eight years, traumatic causes, situations that could be hazardous, or calls from health care facilities. The VFR can accept or reject the mission. Every VFR who accepts the mission receives a map with directions in the application, along with instructions to either go directly to the scene or bring a publicly available AED first and then proceed to the scene. The VFR themselves decide whether to bring the AED or instead go directly to the scene. In this region, up to 40 VFRs, depending on whether there are so many VFRs available within a radius of 10.0 km, can be located and dispatched each time. More detailed information about the number of alerted and accepting assignments as well as distance for the first VFR to arrive at the scene, is available in Svensson et al. [26]. After the mission, a web link to an online user survey is sent via e-mail and text message to all VFRs who had accepted the mission, and they were asked to answer a set of questions related to that particular mission.

### Recruitment and participants

Participants were recruited using a purposive sampling strategy [23] to gain a heterogeneous sample of VFRs dispatched in each of the eight municipalities in the studied region. The inclusion criteria were as follows: a) the participant must be active as a VFR and using SMS-lifesaver, and b) they must have been dispatched and have accepted a mission as a VFR to OHCA at least twice in the last18 months. Heartrunner user surveys were used to identify presumptive participants. Participants were recruited with help from a contact person at Heartrunner, who forwarded an information letter about the study to presumptive participants who were dispatched as VFRs to OHCA and who answered the greatest number of user surveys in each of the eight municipalities. If the presumptive participant was interesting in taking part in the study, they were asked to contact the authors. The last author contacted the interested participants, provided detailed information about the study, and booked a time for the interview. In total, 33 presumptive participants were invited and 18 showed an interest in participating. At the time of the interview, two of the participants could not be reached and the interpretation was that they no longer wished to participate. The final sample consisted of 16 participants, presented in Table 1.

**Table 1:** Characteristics of the participants

		**Voluntary first respondents** **(*n*=16)**
**Age (years)**		
Mean		49
Median		50
Range		23–67
	18–30	1
	31–40	2
	41–50	5
	51–60	5
	61–70	3
**Gender**	Women	5
	Men	11
**Education**	Secondary level	2
	High school level	10
	University level	4
**CPR**^**a**^** training**	Yes	16
	No	0
**Professional health care or rescue education**	Registered nurse	3
	Auxiliary nurse	2
	Police officer	1
	Firefighter	3
	No	7
**Completed missions (*n*)**	1–2	0
	3–4	6
	5–8	6
	9- more	4

^a^*CPR* cardiopulmonary resuscitation

### Data collection

As the data collection was performed after the height of the COVID-19 pandemic, participants were given an opportunity to choose between face-to-face interviews or video interviews [27]. One face-to-face interview (conducted in the participant’s home) and 15 individual video interviews using Zoom were conducted by the last author between March and June 2022. Interviews started with an overarching question: “Could you please tell me about your experiences of being dispatched as a VFR to suspected OHCA? Please base your narratives on real situations”. Probing questions, such as “Can you explain?”, “Can you tell me more?”, and “Could you please give me an example?” were used to support the interview process and encourage narration. One pilot interview (included in this study) was conducted to evaluate the validity of the overarching question. The interviews were audio recorded and covered in total 951 min of data (mean time 59 min ranging from 30–90 min) and were transcribed verbatim by the first and last author.

### Data analysis

Data were analysed based on Elo and Kyngäs’ [28] description of qualitative content analysis. As prior knowledge about VFRs’ experiences of being dispatched to suspected OHCA is still fragmented, an inductive approach was used. An overview of the analysis process is presented in Table 2.

**Table 2:** The analysis process—overview

The analysis process	Authors’ responsibility in the analysis process
The analysis began with several individual open-minded readings of the transcripts to obtain a general understanding of and become immersed in the data	Performed independently by two authors (CA, EKA)
In the next step, the transcripts were abstracted into codes through writing notes and headings in the margins of the transcripts while reading them	Performed independently first by two authors (CA, EKA), who then get together and discussed the codes before compiling them into a coding sheet
The codes were transferred into tables, and grouped according to similarities and differences, with a focus on the aim of this study	The first author (CA) took the lead in the analysis, the last (EKA) acted as co-analyser
Through interpreting similarities and differences, further abstraction was achieved, resulting in six generic categories including sub-categories. As a final step, generic categories were grouped and once more reduced to a main category representing the abstracted results	The first author (CA) took the lead in the analysis, the last (EKA) acted as co-analyser
Finally, the other authors (BN, AS) validated the analysis by reading the transcripts and taking an active part in discussing the interpretation of the findings	All authors (CA, BN, AS, EKA)

## Results

**Fig. 1:**
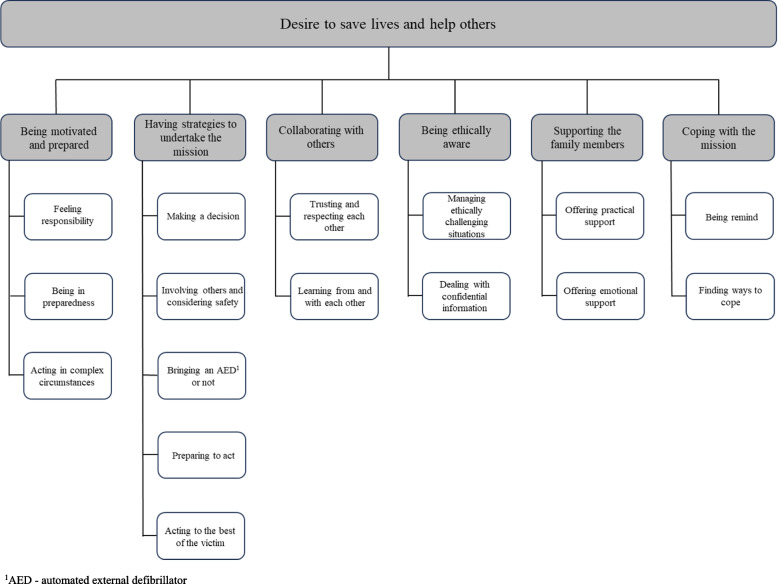
Overview of the findings; main category, generic categories, and subcategories

VFRs’ experiences of being dispatched to suspected OHCA were concluded in the main category, ‘Desire to save lives and help others’. The results showed that VFRs had a genuine desire to contribute to saving lives in a rural area experienced as especially vulnerable. Regardless of the circumstances, they were prepared to leave everything behind and act for the best for the victim and their family members. In their missions, they were guided by ethics when they acted in complex and challenging circumstances. In the presentation of the results, sub-categories (see Fig. 1) are interwoven in the description of the generic categories.

### Being motivated and prepared

Being dispatched as a VFR in a rural area awakened a sense of responsibility for one’s fellow human beings. VFRs described a genuine will to help and felt proud to be a VFR. A need to back each other up was described due to the knowledge that it could take a very long time before the ambulance would arrive to the scene. Other motivational reasons to be a VFR included personal experiences and a wish to use their education and experience in CPR to contribute to the community. The possibility to be involved and help save lives was perceived as leading to feelings of ‘satisfaction’, ‘well-being’, and ‘pride’. However, not being able to accept a mission could lead to ‘feelings of guilt’, and disappointment was described when a VFR arrived on the scene and was not needed. Despite this, they expressed a motivation and will to continue to serve as a VFR.

“The motivation is simply to be able to make an effort to help my fellow human beings or your fellow human beings and with the competence and experience I have, it feels good to share, and it is, even if it doesn't always go the way you want when you get out, it still feels good, but I was there, I took care of the victim, I took care of the family members afterwards…I would be extremely frustrated if I knew that 100 m from home someone collapsed and had a cardiac arrest, and no one did anything.” [Participant 2].

Being a VFR required an awareness and preparedness that the victim could be someone you know, a close friend, a work mate, or even a relative. In rural areas, everyone knows each other, which could mean even upon first seeing the address on the mission, they could assume they knew to whom they were going. It was experienced as emotionally challenging to perform CPR on a victim they knew, but knowing the victim could also be an advantage, as they could support the family members, both during the OHCA and later on.

“Then I got the mission on my mobile phone and my wife looked at the phone and she said the address which was up here in village on the xx street where we have our friends and then I said it must be at the home of (name of the friends) …because you live in a smaller village, you know quite a lot of people here, so there is a big chance you will arrive to someone you know… I'm leaving to do something useful, I can't go out and think, if it's my old work friend or something like this, you're so focused on what you want to do.” [Participant 8].

VFRs expressed a need to be prepared to act in complex circumstances, including ones involving blood, violence, nakedness, unpleasant smells, and sounds. Sometimes they were required to take care of hysterical family members and frightened children. VFRs felt that they also needed to be prepared to manage situations other than OHCA, such as people affected by alcohol and people with panic disorders. They experienced that COVID-19 pandemic totally changed the circumstances of the mission, but most VFRs were ready to risk being infected, even before the mass vaccination began. To increase preparedness, some VFRs bought a kit with gloves, hand hygiene gel, and a pocket mask to use when dispatched.

“The first thing you should think about when you enter the door so when you don't know how people react, that you are clear and introduce yourself and tell them why you are there, I'm here because of a cardiac arrest or I'm a VFR…I usually try to say it before I enter the apartment, because otherwise it can get, it can get very crazy and wrong, even if you are there to help.” [Participant 7].

### Having strategies to undertake the mission

The participants described different strategies to undertake the mission. The decision to accept or reject the mission depended on conditions related to local knowledge, previous experience, and personal circumstances. Local knowledge affected decisions through understanding the infrastructure and the service in the municipality of this region (if firefighters are available full-time or part-time, whether there is access to FIP, and the distance to the nearest ambulance and/or hospital). The distance to the scene was described as crucial in whether to accept or reject the mission and long distance and poor local knowledge could lead to rejecting the mission, as VFRs were aware of the importance of time in delivering CPR. VFRs described the application as unreliable, since the distance to the scene in real life was sometimes much longer compared to what was shown before accepting the mission. A possible improvement could be to get the address directly in the application. Local knowledge affected the strategies guiding which missions to accept, and missions out in rural areas were prioritised. They experienced considerably fewer VFRs arriving the scene in rural areas and sometimes they were completely alone. They wanted to know how many VFRs had been dispatched and how far away the ambulance was, which together influenced their strategies in undertaking the mission. Other suggested improvements were to be able to check whether the application is working correctly and to choose which times of day the application would be active, since VFRs wanted to receive missions, even at night.

Personal circumstances that affected their decision could include whether one had someone who could take care of one's children or access to suitable vehicle, depending on the distance to the scene. VFRs stressed the importance of not being tired when arriving to the scene, which often resulted in taking the car despite the proximity of the scene. Another thing affecting whether to accept or reject missions was whether VFRs were at work. Some VFRs could accept the mission at work, as their employers had a positive attitude toward VFRs.

“If it is inside the smaller village, then I will not drive, then you can say no, because I understand in advance that the firefighters are in that village and there is often an ambulance stationed in that village too, so I see no reason to drive there…the smaller village has no ambulance on site…I always drive there…I don't think there is a fire department on site…so I feel that it is more important to drive there and then above all, the most important thing is, I live in the middle of the forest…if there would be anything around here where I live…if there were to be a mission out in the woods where we live, then I might need to help for real and the same in the small village, I might be able to get there first.” [Participant 3].

VFRs sometimes involved their own family members or other persons close to them in their strategies to undertake a mission. This could mean getting help with practical things so as to be able to leave as fast as possible, to drive, or to help find the right way to the scene. Driving to the scene could be described as a ‘fight’ between driving fast and driving safe, both of equal importance. Thanks to their local knowledge, VFRs knew where to drive faster and where to slow down, but it could still be challenging to both drive and navigate by map. Therefore, voice guidance was suggested as a potential improvement. When arriving at the scene, parking the vehicle was an important part of security to ensure accessibility for ambulance personnel and firefighters.

“My family…know exactly what applies…someone disconnects the electric car, someone takes out my car keys while I'm putting on my shoes, it becomes a chain reaction, everyone knows what to do…the daughter went out and disconnected the car, we all helped each other.” [Participant 5].

VFRs described different strategies regarding whether to bring an AED to the scene. Their decisions depended on their local knowledge (knowing where to find an AED, if it was in a locked place, knowing that the FIP is standby, or whether firefighters are stationed near the scene and always have access to an AED). They were determined to go directly to the scene to commence CPR and not ‘waste time’ by bringing an AED. The only exception was if the AED was on the way to the scene; then no time was lost. The decision to not bring an AED led to questions about whether commencing CPR or getting the AED was more important. VFRs described situations where they could not get the AED because it was under lock and key, or they were not allowed to take it as neither the VFR nor application were known. This caused frustration and led to reluctance bringing an AED next time. The suggested improvements were having several available AEDs in rural areas, the ability to unlock the AED-locker with the application, and to further develop the application so it can alert the VFR when they are driving close to an AED.

“It's just one tool in the toolbox, the AED, the other tools are chest compressions and mouth-to-mouth ventilation…and it's not always possible (time) to bring up these public AED…and then I know that the firefighters are alerted to OHCA and medical missions, but not everyone is aware of it …and then not all places (municipalities) have part-time FD or full-time FD at home either, it may be a long drive before they are there, and it would be a little easier if there had been AED in more places.” [Participant 2].

On the way to the scene, the VFRs mentally prepared themselves to act. They wondered whether it would be an OHCA and whether the environment would be safe. They described how they mentally repeated the CPR logarithm. The possibility of contacting an emergency medical dispatch centre to get support and advice when needed on the scene was described. The VFR suggested that the application should provide information about security and changed circumstances; e. g. when CPR is no longer needed. Introducing themselves as VFRs and asking if help is needed was important when arriving at the scene, as this gave them legitimacy to act.

“When you are going to the mission, you are fully focused on commencing CPR. I rehearse 30:2 a couple of times before I arrive and try to slow down my breathing because you can be quite breathless in such a situation when you know that it is minutes, so you take a few deep breaths, so you don't seem stressed when you come to the person in need.” [Participant 1].

Acting to the best of the victim was the priority at the scene. When first arriving, the VFRs began by assessing the victim and, if needed, commencing CPR as quickly as possible. When several VFRs already were on the scene, it was described as important to ensure the quality of ongoing CPR and either correct it or switch roles with the other VFRs and sometimes also act as a leader. When there were other VFRs, ambulance personnel, or firefighters involved, some VFRs chose to turn back immediately, while others decided to stay or leave depending on whether there were enough resources on hand. If there only was one ambulance, they knew that they could need their help. Some VFRs always asked if help was needed, even when there were two ambulances, while others just turned back. The VFRs encountered some barriers to reaching the victim like locked doors, not knowing the port code, dogs at the scene, and, in a larger building, not being able to find the victim.

“Then it's important to commence CPR, everything else is uninteresting at that moment, unless it's someone, what can you say, they're lying there with electricity in them or they're lying in a bathtub…full of water… then you have to do another action first, but when you getting close (to the scene), the first focus is to commence CPR as soon as possible…that's the only thought you have in that situation. Then everything else can come afterwards, so there is always full focus on the patient.” [Participant 4].

### Collaborating with others

VFRs described mostly good collaboration with others involved in the mission. This collaboration was characterised by trust and mutual respect, which was described as necessary in unpredictable situations, as every link in the ´chain of survival´ was experienced as equally important. Participants emphasised the importance of effective communication within the team (i.e., other VFRs, ambulance personnel, firefighters) and a lack of prestige in the collaboration, which made it natural for the VFR to take a step back when persons with higher competence arrived.

“When they (ambulance personnel) came I continued with the CPR because they tell me to continue with compressions, and I continue and they start connecting LUCAS, someone prepares drugs, and someone takes care of the wife and so on, after a while when they feel they have the situation under control, they take over.” [Participant 11].

Good collaboration at the scene was described as an opportunity to learn from and with each other, in order to learn how to act more appropriately next time. However, situations were described where the VFRs experienced being ‘pushed aside’, which led to feelings of not being good enough, and where they were pressured, by the ambulance personnel, to answer questions they were not able to answer.

To further develop knowledge, competencies, and collaboration, joint training and educational meetings for everyone included in the ´chain of survival´ were suggested. This would be an opportunity to learn through sharing experience and knowledge, and even a way to prevent the feeling of being ‘pushed aside’.

“Perhaps to somehow organise large meetings for VFRs, maybe some training…I think it would have been valuable…given even more, better quality…That it perhaps includes both information or training or something like that, from experienced and talented people, and that you meet together and talk to each other, exchange experiences, it could also be that ambulance personnel and firefighters might have some input… perhaps great knowledge could be exchanged between those active in the profession and VFRs.” [Participant 14].

### Being ethically aware

VFRs described how they had to face and manage ethically challenging situations at the scene. One challenging situation described was to decide whether to commence CPR when the victim was assessed as obviously dead upon arrival at the scene and when the VFR felt that CPR would be futile. Some VFRs refrained from CPR due to ethical reasons, while others always commenced CPR as they felt the decision was not theirs to make. Not commencing CPR on an obviously dead victim was described as an act of respect for both the victim and the family members, as it would raise false hope in the family members, which was experienced as unethical. Sometimes they had to act against their own beliefs when the VFR decided not to commence CPR and the firefighters made the opposite decision. Among VFRs who always commenced CPR, this sometimes led to remorse. Another ethically challenging situation described by VFRs was being dispatched to a victim who has a palliative diagnosis. In those cases, it was important to listen to the family members and respect their will.

“We can't state that she's dead of course, but there, out of pure respect, I let her be…if it's been this long, I'm not going to start struggling with that poor aunt, when her son is standing there... I'm not allowed to say anything like she's dead or that, but out of pure respect for him and for her, then I don't start tearing into someone who's been lying for so long, I will not do it, even if, even if everyone says you are not allowed to (not commence CPR)…but it's personal ethics, it's like, it becomes completely grotesque to start doing something there.” [Participant 6].

Rejecting a mission was another ethically challenging situation. VFRs only rejected a mission when it was impossible for them to go, but despite this, it could lead to remorse, even in cases where they could explain their decision logically. Sometimes VFRs had to cancel a mission, after first accepting it, when the distance to the scene turned out to be too far. This was described as ‘cancelling a contract’ and led to thoughts about the victim, thoughts that someone may die because of their decision, and their lack of ability to accept that mission. All of this could lead to negative emotions and thoughts about the victim, how the victim fared, and if the situation would have turned out another way if they had had the ability to act.

VFRs expressed that during the mission, they sometimes received confidential, personal information about the victim and their family, that they otherwise would never have received. This could lead to feelings of exposure among the family members that the VFRs needed to manage. They conveyed the importance of being discreet and not talking about the mission with others. Some of them talked with their own family, while for others it was totally out of question. Participants expressed the importance of preserving integrity for the victim and their family members, as information in rural areas can spread quickly.

“This first (mission) I went to, she (the victim) was already dead, you think about it because you know the relatives, you usually have some kind of relationship with them. When you get home, you should not talk about it with your own family, you should not mention who you went to until it becomes official that the person is dead…I try to be quiet in any case, it can be a bit difficult not being able to talk about it…thoughts come up in my head, did I do the right thing…it's not stated anywhere in the app, but for me discretion is important…I think it is very important that you don't talk about it widely, especially not in such small villages, it is not in my mind that you would start talking about it.” [Participant 10].

### Supporting the family members

Supporting family members was seen as a natural part of the mission and concerned both practical and emotional support. Practical support included taking care of children, giving information, informing other relatives, giving a ride to the hospital, and explaining the situation. This support sometimes also involved making contact with other important persons for the victim’s family members; e.g., school counsellors or social services in the municipality.

“I mean it's traumatic when your husband is affected, for his wife who has to stand next to him and watch, she needs all the comfort in the world and it's not certain that she will be allowed to come with the ambulance either and it may be that she will come along, but she can't get home, so you have to stand up for the person who is next to her, you may have to call children and so they will find out, it has to happen, the ambulance staff can't handle that part, they have to save the person in question in the first place.” [Participant 12].

The emotional support adapted to the situation could include distracting, calming, offering a sense of safety, and staying at the scene as long as the family members wanted them to, even after the ambulance had left. VFRs described the importance of listening to the family members and respecting their decisions if they wanted to stay next to the victim during CPR or if they wanted to leave the room. In conversation with the family members, they chose their words carefully, as they did not want to evoke false hope. When the VFR was known to the family members, it could mean that they were forced to manage complicated situations such as conflicts within the family. Leaving one´s name and phone number was a way to give the family members a possibility to make contact. Someone even tried to return to the family members the day after, to support and offer answers to questions.

“Then the ambulance came and then it became natural that I took that role and talked to her (the victim’s wife) and explained a little what the ambulance personnel were going to do and asked if she had someone she wanted to call, I took on that role instead…as a VFR you can do other things around, as in this case, taking care of a family member or other things around, especially when you are in a rural area…in a city, you are close to everything but here you have a long way to the ambulance, so I feel a greater responsibility to go to those missions, a greater commitment to go to these missions than if it were in the city, where you can easily have a feeling that someone else can do it, but here I feel maybe it's just me, maybe there's no other VFRs around so you feel a bigger…that maybe it depends on me, in a positive meaning, that I can really make a difference.” [Participant 15].

### Coping with the mission

VFRs described uncertainty as to whether the victim survived and a desire to know how the victim was doing. Although they sometimes tried to get this information from others, it was difficult, as it brought relief and happiness if the victim had survived and powerlessness and sadness when the victim died. The positive emotions were enhanced when the VFR met survivors in the community. The negative emotions could linger for a long time, and they were reminded of them every time they met the family members.

Most VFRs were aware of the possibility to receive support in the region but did not feel that they needed it. However, they emphasised the importance of debriefing to be able to leave the mission behind. They wanted to know whether they could have done something different in a particular situation. Different ways of coping were described, such as talking with others engaged in the mission, talking with one’s own family, or debriefing together with the firefighters. They expressed that the trust and safety they experienced within the chain contributed to coping as they often know each other in the rural area. Some of them chose to process the mission by themselves, even if it could take some time. Returning to the victim’s family members was also described as a way of coping with the mission.

“We had each other's support…we sat and talked about what had happened…everyone did what they had to, gave everything all the way then it is, I think, if things come up, well, let it, I'm not afraid of having flashbacks, or other things that come like discomfort, that’s the way it is…it may come when it comes, it's like a normal crisis, it appears less and less.” [Participant 13].

VFRs suggested that a way to both support each other and to improve the ´chain of survival´ was to arrange meetings between all involved. The meetings could include team lectures, e.g., crisis and coping, CPR training, practical training together with firefighters, research updates, and opportunities to share knowledge and experiences with each other. Those meetings could be a safety net, a way to find those who needed more support.

“There is no possibility that the healthcare system should be able to follow up on all small missions and call them and ask how they are, but on the other hand, you could arrange, perhaps twice per term, arrange some kind of reconciliation lecture…that thing with coping mechanisms and how to handle a crisis and create an understanding, just such a thing, so that you might have a little basic discussion where you can talk and air some thoughts, and experiences, maybe it's enough to feel like you're not alone, even if this is damn uncomfortable.” [Participant 7].

## Discussion

The main result of this study has been presented in the main category: ‘*Desire to save lives and help others*’*,* demonstrating the VFRs’ sense of responsibility and determination to contribute to their fellow human beings and to their community of this rural area. The VFRs’ desire to help was also described by Södersved Källestedt et al. [21], which indicates the potential of VFRs to contribute in the first link of the ´chain of survival´ increasing victims’ chances of survival. Representatives from ambulances together with representatives from municipalities and regions should take advantage of VFRs’ desire to save lives and support them in that mission. This is particularly important in rural areas, where the ambulance response time can be prolonged, and VFRs need to act for longer periods of time, sometimes completely alone.

The results showed that the VFRs experienced emotionally- and ethically challenging situations at the scene, such as deciding whether to commence CPR when the victim is assessed as obviously dead and when they felt CPR would be futile. Previous research [21, 29] also shows that VFRs experience challenging and distressing situations at the scene, where difficult decisions need to be made rapidly; e.g., regarding whether to commence CPR. Ethically challenging situations associated with commencing, withholding, or terminating CPR in connection with OHCA missions have been previously described from the perspective of ambulance personnel [30, 31]. Andersson et al. [32] showed in a systematic review that such decisions may be difficult and a lack of consensus between resuscitation providers may occur. It is important to remember that ambulance personnel have education and training that promotes decision-making and ethical reflection. According to Andersson et al. [31] they can get help from senior colleagues either on scene or via phone. In addition, they can lean on both national [33] and international [4] resuscitation guidelines which VFR do not have. Systematic training and support directed at VFR is lacking in the Swedish context. A European consensus conference [34] concluded that VFRs should be systematically prepared, as it can help alleviate the acute psychological stress that some VFR may experience. Therefore, offering the possibility to gain access to VFR follow-up programmes is important; similar to Rolin Kragh et al.’s [35] description of VFR who wish to participate in defusing/debriefing meetings and in that way process their experience and, contribute to sustainability increasing retention. In a study [36] about motivational and demotivational factors to act as a volunteer in emergencies, volunteers without professional experience described feeling more burdened after alerts and alarm fatigue. This could be a reason to leave the VFR assignment, and participating in a follow-up-programme could help prevent this. On the other hand, volunteers from rural areas or small cities described a high emphasis on community, helping others, and helping their community [36], which is in line with what the VFRs in this study expressed. Further research is needed to determine whether support interventions, especially in rural areas, could help VFRs deal with emotionally and ethically challenging situations at the scene.

The results showed that the VFRs’ desire to help not only included the victim but also supporting the victim’s family members both in emotional and practical ways. They expressed the importance of effective communication, of calming the family members, and of being ready to stay with the family members for a while after the ambulance had left, and even left their phone number or contacted the family member afterwards. This is in accordance with the findings of Nord-Ljungqvist et al. [37], where receiving clear information and calm communication from firefighters contributed to feelings of safety and trust, and staying and talking with the family members about the situation led to feelings of comfort [37]. Although VFRs do not need to be professionals, they could contribute the same feelings of comfort to the family members of OHCA-victims. This is described by Rolin Kragh et al. [38], where family members expressed relief regardless of whether a professional or a VFR arrived at the scene. In our study, VFRs also offered practical support to family members, such as giving information and taking care of children. Rolin Kragh et al. [38] describes that such seemingly small things gave the family members of the OHCA victim a feeling of been taken care of and reduced feelings of loneliness; however, this study did not include any missions where the family member and the VFR knew each other, as were described in our study. When family members and VFR know each other, which is more common in rural areas, family members seem to ask for more complex help, which may be a greater challenge for the VFRs. Due to this, it could be even more important to offer possibilities for education and debriefing for VFRs active in rural areas regarding the complexity of a VFR mission. VFRs in our study described leaving their phone number or contacting family members the day after the OHCA. The possibility to contact the first responder (firefighter) afterwards could help fill in the knowledge gaps and contribute to a sense of control. The way the VFRs acted in this study, may contribute in the same way to a sense of control for the family members [37, 39].

VRFs ask for the opportunity for collaborative training, more educational meetings, and the ability to share knowledge and experiences with other stakeholders involved in the ´chain of survival´. Often, they feel included as a part of the team at the scene, but also emphasise sometimes feeling left out and excluded when going to a scene by themselves and being ‘pushed aside’ by arriving ambulance personnel. The result is in line with other lone working FRs, such as FIP [40] and RNs [39, 41] that states missing both preparatory conversations on the way to the scene and debriefing after the incident. Also, receiving information about the outcome of the patient is desired, which is in line with what other VFRs have stated as important [39, 42]. Providing collaborative training to VFRs and other stakeholders collaborating at the scene, can contribute to achieving minimum qualifications, preventing psychosocial distress and, strengthening the VFRs in their missions, all together resulting in increasing the VFRs safety, which is requested by researchers [43, 44].

### Methodological considerations

In qualitative design studies, trustworthiness can be evaluated using the concepts of credibility, confirmability, dependability, and transferability [45]. The sampling strategy was deliberately aimed to achieve variation, and the sample was heterogeneous regarding participants' age, education, number of completed missions, and represented each of the eight municipalities in the region. The sample size of 16 VFRs is small, however the interviews reflected depth and richness in a large amount of qualitative data, as the participants were experienced VFRs, and nearly half of them had professional healthcare- or rescue education. In the recruitment process, VFRs who answered the greatest number of user surveys were contacted, which could have led to selection bias. However, at the same time, it was important to gain access to experiences of being dispatched to OHCA, which the included VFRs had. A limitation is that the voices of less-experienced VFRs are missing, which could be of importance in the development of appropriate support. The VFRs in Sweden are only alerted from 7 a.m. to 11 p.m., which differs from some other international systems where VFRs could be alerted around the clock. This could be a limitation, as the experiences could be different when VFRs also get alerted during the night. Data were analysed in collaboration with the team, resulting in triangulation between all authors during analysis, ensuring confirmability. Quotations from different interviews were provided to illustrate participants' personal experiences. To attain confirmability in the analytical process, authors were vigilant, reflective, and self-aware as per their previous experience so as to not allow them to unconsciously steer the process of analysis. Transferability was facilitated through detailed descriptions of the context, and the participants' characteristics as well as data collection and analysis processes were described in detail, offering the reader the ability to follow the different steps in the research, facilitating reader assessment of transferable findings. Including participants from only one region can be seen as a limitation, as VFRs’ experiences could be affected by the organisations’ structure and the culture of a specific region; however, VFRs systems are used in several countries and the ´chain of survival´ is internationally acknowledged, which could increase transferability.

## Conclusions

VFRs need to be seen as an important link in the ´chain of survival´. Those acting in rural areas have a desire to help others, and express feelings of responsibility to society, but above all, feel responsible for the people living in their neighbourhood. The results show that VFRs contribute to a sustainable society, both economically, through saved lives, and socially, by building trust, as they often are known in society. Being a VFR in a rural area means facing emotional and ethical challenges, as they more often know the victim and/or their family and are also known by them. To maintain the will to be a VFR and reduce the risk of poor health in this group, it is important to develop ways to support VFRs before, during, and after the mission.

The results show that VFRs in rural areas more often face a long distance to the scene and when leaving everything to take on the mission, their social networks also are included and engaged. Further development of the distance calculation and clearer instructions in the application could contribute to less stress and remorse among VFRs. When the VFRs were ‘pushed aside’ by ambulance personnel or firefighters during the mission, the VFRs had a sense of not being a part of the team. Developing meetings including all parts of the chain, including VFRs, could contribute to increased knowledge and understanding of each other´s roles at the scene, resulting in a better-functioning team, which can contribute to increased patient safety. Both previous research and the results of this study highlight the need to also support VFRs after the mission. It is difficult to determine who is responsible for developing this support, however, VFRs must be supported. As VFRs in rural areas experience both emotional and ethical challenges, support for this group should be prioritised. Designing, implementing, and evaluating support interventions is necessary, especially in rural areas, as it can contribute to more people becoming and remaining VFRs, which could contribute to a sustainable development. This study investigates only the VFRs perspective, and it is obvious that they collaborate with several stakeholders, all of them important in ´the chain of survival´. Further research should explore the collaboration and the experiences of all stakeholders included in OHCA.

## Data Availability

The data generated during the current study are not publicly available, as the nature of qualitative data is difficult to fully anonymise. The participants in this study were not asked to agree to their data being publicly available. However, all the aggregated datasets in Swedish are available from the corresponding author on reasonable request.

## Abbreviations

AED: Automated external defibrillator
BLS: Basic life support
CPR: Cardiopulmonary resuscitation
EMS: Emergency medical services
FD: Fire department
FIP: First incident person
FR: First responder
LUCAS: Lund University cardiopulmonary assist system
OHCA: Out-of-hospital cardiac arrest
RN: Registered nurse
VFR: Voluntary first responder
